# FUNCTIONALITY OF UPPER GASTROINTESTINAL CANCER PATIENTS WHICH HAVE UNDERTAKEN SURGERY IN HOSPITAL PHASE

**DOI:** 10.1590/0102-672020180001e1353

**Published:** 2018-06-21

**Authors:** Epamela Sulamita Vitor de CARVALHO, Ana Cristina Machado LEÃO, Anke BERGMANN

**Affiliations:** 1 Instituto Nacional de Câncer - INCA (National Cancer Institute, Rio de Janeiro, RJ, Brazil

**Keywords:** Gastrointestinal neoplasms, Postoperative period, Immobilization, Muscle strength, Neoplasias gastrointestinais, Período pós-operatório, Imobilização, Força muscular

## Abstract

**Background:**

Cancer patients present various physiological, metabolic, social and emotional changes as a consequence of the disease’s own catabolism, and may be potentiated in the gastrointestinal tract cancer by their interference with food intake, digestion and absorption.

**Aim: T:**

o evaluate the functionality of upper gastrointestinal cancer patients which have undertaken surgery and analyze the factors associated with changes in strength and functionality during hospitalization time.

**Methods:**

Prospective analytical study in patients with cancer of the upper gastrointestinal tract which have undertaken surgery. Was evaluated the handgrip strength using a hand dynamometer and functionality through the functional independence measure and Functional Status Scale for Intensive Care Unit in the preoperative period, 2^nd^ and 7^th^ postoperative day.

**Results:**

Were included 12 patients, 75% men, and mean age was 58.17 years old. The most prevalent tumor site was stomach (66.7%). There was a progressive reduction from the pre-operative palmar grip strength to the 2^nd^ and 7^th^ postoperative day, respectively. There was a decrease in functional performance from the preoperative period to the 2^nd^ and a gain from the 2^nd^ to the 7^th^ postoperative day (p<0.001).

**Conclusion:**

An important reduction in the handgrip strength and functionality was evidenced during the postoperative period in relation to the basal value in the pre-operative period.

## RESUMO

**Racional:**

Os pacientes com câncer apresentam diversas alterações fisiológicas, metabólicas, sociais e emocionais como consequências do próprio catabolismo provocado pela doença, podendo ser potencializadas nos canceres gastrointestinais pela sua interferência na ingestão, digestão e absorção dos alimentos.

**Objetivo:**

Avaliar o comportamento funcional de pacientes com neoplasia gastrointestinal alta submetidos ao tratamento cirúrgico e analisar os fatores associados às alterações da força e funcionalidade durante o tempo de internação.

**Métodos:**

Estudo prospectivo analítico de pacientes com câncer do trato gastrointestinal alto submetidos ao tratamento cirúrgico. Foram avaliadas a força de preensão palmar por meio do dinamômetro manual e funcionalidade por meio da Medida de Independência Funcional e *Functional Status Scale for Intensive Care Unit* no pré-operatório*, no* 2º e 7º dias do pós-operatório .

**Resultados:**

Foram incluídos 12 pacientes, 75% homens, com idade média de 58,17 anos. O sítio tumoral mais prevalente foi o estômago (66,7%). Houve redução progressiva da força de preensão palmar das fases pré-operatória em relação ao 2º e 7º dias do pós-operatório, respectivamente. Ocorreu diminuição do desempenho funcional do pré-operatório para o 2º dia e um ganho do 2º dia pós-operatório para o 7º dia (p<0,001).

**Conclusão:**

Houve importante redução na força de preensão palmar e funcionalidade durante o período pós-operatório em relação ao valor basal no pré-operatório.

## INTRODUCTION

Cancer patients present various physiological, metabolic, social and emotional changes as a consequence of the disease’s catabolism, that may be potentialized in the gastrointestinal tract cancer due to its interference in food intake, digestion and absorption. These changes may lead to several consequences and may be aggravated when associated with treatments, which include surgical resection, chemotherapy and radiotherapy, staging of the tumor and the affected organ^4^ ^,^ ^12^ ^,^ ^13^.

Among the treatments, the surgical approach is regarded as essential to the curative treatment^11^ ^,^ ^13^. Among the complications of the surgical procedure, the pulmonary complications are one of the most frequent after abdominal operations and are also responsible for the increase in morbidity and mortality due to the longer period of hospitalization and health-related costs^26^. The complications occur more frequently after procedures in which the surgical incision is made above the umbilical scar^17^, a fact also acknowledged in bariatric patients^20^ ^,^ ^28^. Its incidence is related to the existence of preoperative risk factors^5^ and also to perioperative factors, such as anesthesia, central nervous system depression, use of invasive mechanical ventilation, ineffectiveness of cough, pain and immobilization^9^ ^,^ ^17^. This situation reduces cardiorespiratory capacity, that is also negatively influenced by postoperative physical inactivity, generating loss of muscle strength and deconditioning^30^.

The functionality can be understood as the ability of the individual to perform certain activities or functions that influence the simple and the complex behaviors required in daily life. Functional impairment incapacitates the individual to perform basic activities, such as taking care of himself and his surroundings independently^29^. Thus, it is important to know the level of functional capacity in both short and long term, after the surgical procedure, to enable a better management in health care, which surpasses the clinical solution of the disease, goes until the desired functional recovery.

The aim of this study was to evaluate the functionality of upper gastrointestinal cancer patients who have undertaken surgery and to analyze the factors associated with changes in strength and functionality during the hospitalization period.

## METHODS

Prospective analytical study performed in the section of abdominopelvic surgery at the Hospital do Câncer I in the Instituto Nacional de Câncer (INCA), Rio de Janeiro, RJ, Brazil. It was approved by the Research Ethics Committee of the INCA under the opinion of number 1.650.497.

In this study were included patients older than 18 years without cognitive impairment and who agreed to participate through the free and informed consent term. The patients with neuromuscular diseases, those who underwent exploratory laparotomy and those who remained for more than 48 h in the intensive care unit and postoperative unit were excluded. After agreeing to participate and sign the consent form, the sociodemographic, clinical and therapeutic aspects were obtained through an evaluation sheet by means of an interview and the medical record systems of the hospital unit. Then, the participants were submitted to a physical activity questionnaire, pain scale, functional scales and performed a peripheral muscle strength test. They were evaluated in three moments during the hospital stay: preoperative (one day before surgery), 2^nd^ and 7^th^ postoperative day (POD).

The physical activity evaluation was made through the international physical activity questionnaire (IPAQ), version 8, validated for the Brazilian population^13^. The version used was the short form, that includes an interview approach to perform the preoperative evaluation, containing questions about the frequency and duration of physical activity over the last week, which classifies the individual into categories: very active, active, irregularly active and sedentary. In order to analyze the data of the physical activity level, the IPAQ was used in an adapted form, in which the patients were classified into two groups: sedentary and non-sedentary (very active, active, irregularly active).

The pain was measured using the visual analogue pain scale (VAS). It consists of a 10 cm line, in which the left end, the zero, indicates absence of pain and the right end, the 10, indicates the worst pain imaginable^16^. This ruler was presented to the patient to identify and classify his level of pain at that moment, by marking a vertical trace on the line. The VAS values were collected in the three stages of evaluation of all individuals.

The functionality was evaluated by the Functional Independence Measure (FIM) and Functional Status Scale for Intensive Care Units (FSS-UCI). The FIM is a scale organized by the classification of the patient’s ability to perform an activity vs. the need for assistance of another person or some adaptation. It evaluates the performance of the individual to accomplish 18 activities, relating to the subscales of self-care, sphincter control, transference, locomotion, communication and social cognitive aspect. This instrument scores the information obtained in a scale from one to seven for each item, in which the score one represents the total dependence of the patient and the score seven, the total independence. The total score in the FIM is calculated from the sum of points assigned to each item within the categories. This score is pre-defined by the scale, according to its functionality equivalent, with a minimum score of 18 and a maximum of 126, in which the largest scores represent better functionality and less dependence^23^.

The FSS-ICU is a scale similar to FIM, but it includes more appropriate activities regarding mobility and transfer in a hospital environment. It includes the evaluation of five basic functions (roll, transfer from supine to sitting position, transfer from sitting to standing position, sitting at the edge of the bed and walking). Each function is evaluated using a scale from one to seven, in which a score of one corresponds to the total dependence and seven, to the total independence; it can be graded a total of 0-35 points, considering the highest values related to the greater functional capacity^31^.

It was also performed the manual dynamometry (MD) or handgrip strength (HS) test to evaluate the peripheral muscle strength, which aims to evaluate the functional state of the skeletal muscle using the JAMAR® hand dynamometer. The HS was evaluated in both hands of the volunteer to avoid the dominance effect, using a hand dynamometer and following the recommendations of the American Society of Hand Therapy^1^. The patients included were instructed to sit with their feet flat on the floor, ankles in neutral position, knees bent at 90º, and thighs resting on the seat. The upper limb evaluated remained with slightly abducted shoulder, elbow in 90º flexion, forearm in neutral position between supination and pronation and the wrist between 0-30º of extension and 0-15º of ulnar deviation. After attempting to familiarize them with submaximal force, they were verbally stimulated to perform three maximum isometric contractions for 5 s and the result was registered in kg. Were collected three measurements from each hand, and used the mean value in all of the analysis made.

### Statistical analysis

The collected data were typed in a spreadsheet of Microsoft Excel® software, exported and analyzed in the Statistical Package for Social Sciences (SPSS) software, version 23.0. The variables were described as percentages and relative and absolute frequencies, or as means and standard deviation, depending on the nature of the variable (categorical or continuous, respectively). The statistical test used was ANOVA and Wilcoxon. The level of significance considered for all tests was 5%. The analyzes were performed comparing the different variables of the sample. 

## RESULTS

**FIGURE 1:**
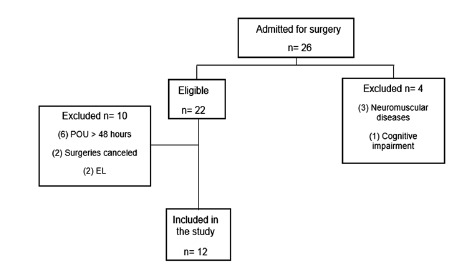
Flowchart of the patients included in the study.

Twenty-six patients were admitted for upper abdominal surgery and 12 of them were eligible and included in the postoperative evaluation (Figure 1).

POU=postoperative unit; EL=exploratory laparotomy

The mean age was 58.17±10.8 years, of which 75% were men. The most prevalent tumor site was stomach (66.7%), followed by duodenum, pancreas, esophagus and liver. The following operations were performed: partial gastrectomies (41.7%), total gastrectomies (33.3%), gastroduodenopancreatectomy (8.3%), duodenopancreatectomy (8.3%) and hepatectomy (8.3%), which presented mean duration of 216.6±57.5 min (Table 1).

**TABLE 1:** Characteristics of the patients included in the study

Characteristics	n	%
**Gender**		
Male	9	75%
Female	3	25%
Smoking		
Yes	2	16,7%
No	8	66.4%
Ex-smoker	2	16.7%
**Ethicism**		
Yes	2	16.7%
No	2	16.7%
Ex-stylist	8	66.4%
**Body mass index (BMI)**		
Low weight	2	16.7%
Eutrophy	3	25%
Overweight	6	50%
Obesity	1	8.3%
Comorbidity		
COPD	3	25%
Cardiopathy	2	16.7%
SAH	1	8.3%
None	6	50%
**Level of physical activity**		
Sedentary	6	50%
Non-sedentary	6	50%
**Tumoral site**		
Stomach	7	66.7%
Duodenum	2	16.7%
Pancreas	1	8.3%
Esophagu	1	8.3%
Liver	1	8,3%
Stage		
I	1	8.3%
II/III	10	83.3%
IV	1	8.3%

n=number; COPD=chronic obstructive pulmonary disease; SAH=systemic arterial hypertension

Regarding the treatment, six (50%) underwent neoadjuvant chemotherapy and all of the patients were assisted by the hospital’s physiotherapy service right on the first postoperative day.

The patients had a mean of 2.66±0.88 physiotherapeutic attendance until the final phase of the collection. The conduct varied between: reexpansive respiratory kinesiotherapy, use of respiratory stimuli, transfer training in bed, seated at the bedside, orthostatism, static gait and ambulation.

The mean length of hospital stay was 12.50±6.1 days. The loss of body weight occurred in 10 patients (83.3%) consisting of a mean of 1.96 kg (p=0.021) until the 7^th^ day after surgery, being a greater loss in patients with chronic obstructive pulmonary disease (p=0.043).

In the EVA evaluation of pain intensity, only two (16.7%) reported mild and moderate pain during the preoperative period. On the 2^nd^ POD, 66.4% reported different pain intensities (mild, moderate and severe); among these, seven (58.1%) maintained mild pain on the 7^th^ POD.

The majority of the patients were right-handed (83.3%). For the evaluation of MD, both dominant and non-dominant hands were used. The dominant hand presented greater muscle strength than the non-dominant, and the men presented greater muscle strength than the women in both hands and in all of the collections (Table 2). There was a progressive reduction of the HS values in both hands, from the preoperative phase, to the 2^nd^ and 7^th^ POD, being statistically significant at the dominant hand (p=0.031).

The relation between MD, age and gender did not present a significant difference. Regarding the clinical variables, the reduction of the MD value of the non-dominant hand presented a significant relation in patients with heart disease (p=0.015). 

**TABLE 2:** Handgrip strength of dominant and non-dominant hand in the sample of gastrointestinal cancer patients

Variables	Dominant hand			Non-dominant hand		
	All	Male	Female	All	Male	Female
	Mean ± SD			Mean ± DS		
PreOP	33.50 ± 8.63	35.7 ± 8.43	26.6 ± 5.68	30.17 ± 9.25	32 ± 9.39	24.6 ± 7.57
2^**nd**^ POD	31.92 ± 7.57*	34 ± 7.07	25.6 ± 6.11	29.33 ± 7.53	31.2 ± 6.35	23.66 ± 9.29
7^**th**^ POD	31.58 ± 7.58*	33.2 ± 7.46	26.6 ± 6.11	27.83 ± 7.33	29.4 ± 6,82	23 ± 7.93

PreOP=preoperative; POD=postoperative day; *p=0,031

**FIGURE 2:**
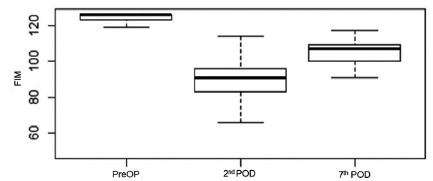
Variation of the functional measures of the FIM during the preoperative period, 2^nd^ and 7^th^ DPO

Through the evaluation of the functionality, using FIM, it was observed that there was a decrease in the values of the scale when the 2^nd^ and 7^th^ POD were compared to the preoperative phase (p<0.001, Table 3), showing a greater variation in the functional measurements between the preoperative period and the 2^nd^ POD (Figure 2).

**TABLE 3:** Comparison of the preoperative period, 2 ^nd^ and 7 ^th^ postoperative days outcomes (Wilcoxon test)

	Pre - 2^nd^ POD					2^nd^ DPO - 7^th^ DPO					Pre - 7^th^ POD				
Variables	Value p	Improvement	Gets worse	Kept	Mean (IC 95%)	Value p	Improvement	Gets worse	Kept	Mean (IC 95%)	Value p	Improvement	Gets worse	Kept	Manteve
VAS	0.06	1	8	3	-1.50 (-2.88 a -0,13)	0.03*	7	1	4	0.41 (-0.61 a 1.44)	0.39	1	6	5	5
HSD	0.03*	2	8	2	-0.33 (-1.67 a 1)	0.5	5	6	1	-1.91 (-3.64 a -0.19)	0.03*	2	8	2	2
HSND	0.4	2	6	4	-1.5 (-3.26 a 0.26)	0.08	4	7	1	-2.33 (-4.85 a 0.18)	0.06	2	8	2	2
FIM	<0.001*	0	12	0	17.75 (9.09 a 26.40)	0.001*	12	0	0	-19.3 (-24.1 a -14.5)	<0.001*	0	12	0	0
FSS-UCI	<0.001*	0	12	0	7 (2.95 a 11.04)	0.003*	12	0	0	-4.75 (-6.98 a -2.51)	0.001*	0	12	0	0

PreOP=preoperative; POD=postoperative day; VAS=visual analogue pain scale; HSD=handgrip strength dominant hand; HSND=handgrip strength non-dominant hand; FIM=Functional Independence Measure; FSS-UCI=Functional Status Scale for Intensive Care Unit; * p<0.05.

ANOVA test with post-test p<0.001; PreOP=preoperative; POD=postoperative day; FIM=Functional Independence Measure.

The FIM scores obtained by sedentary patients with comorbidities and who underwent neoadjuvant chemotherapy were lower when compared to non-sedentary patients, with no comorbidities and who did not undergo chemotherapy; however, this comparison did not present a significant difference between the groups. There was no difference between gender and age in this study.

**FIGURE 3:**
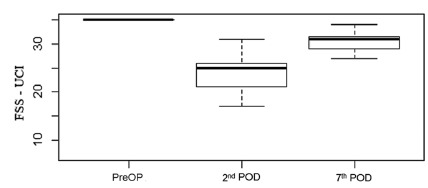
Variation of the functional measures of the FSS-UCI during the preoperative period, 2^nd^ and 7^th^ POD

The FSS-UCI, used to evaluate mobility and transfer, also showed a reduction in its score when the preoperative phase is compared to the 2^nd^ and 7^th^ DPO (p<0.001), showing a greater variation between the preoperative and the 2^nd^ POD (Figure 3). It was observed a significant limitation in the transfer functions during the 2^nd^ POD, in which 10 patients (83.3%) needed the help of a professional or companion to perform decubitus changes and orthostatism. On the other hand, four (33.4%) needed the help of third parties to walk, and five (41.7%) needed some type of supervision and walked slowly. However, there was an improvement in mobility from the 2^nd^ to the 7^th^ POD, when only two (16.7%) patients needed help from third parties to perform the decubitus changes and only one (8.3%), to conduct the march.

ANOVA test with post-test. p<0.001; PreOP=preoperative; POD=postoperative day; FSS-UCI=Functional Status Scale for Intensive Care Unit.

There was a reduction in the FSS-ICU values of the sedentary individuals who underwent neoadjuvant chemotherapy in comparison to the non-sedentary groups and who did not undergo neoadjuvant chemotherapy; however, this reduction was not significant. Regarding the other variables, age, gender and comorbidity, the groups were similar.

## DISCUSSION

In this study, it was observed that there was a worsening of the peripheral muscle strength through MD and functional capacity when compared to the preoperative and the postoperative periods.

It was verified that eight patients (66.4%) reported an episode of abdominal pain during the 2^nd^ POD and the 7^th^ POD, seven of them (58.1%) reported persistence of the abdominal pain regardless of the EVA score. These values corroborate with studies described in the literature that show that approximately 44% of patients submitted to abdominal surgery reported feeling some type of pain during the post-surgical phase^22^. On the other hand, although not verified in this research, it is necessary to consider the data described in the study of Oliveira et al. ^21)^ that shows that 45% had postoperative abdominal pain, indicating a non-effective pain treatment.

The results showed a significant reduction in MD of the studied population, presenting a progressive decrease during the collection phases, with a greater loss when compared to the group with heart disease. Savage et al.^25^ used a sample of healthy and cardiopathy individuals and showed that patients with cardiac disease presented a loss of global functional capacity, affecting the loss of peripheral muscle function, which was aggravated during hospitalization due to bed rest, similar to this sample, in which cardiac patients presented a significantly lower HS when compared to those without cardiac comorbidities.

The reduction of HS is understudied when it comes to surgical cancer patients, but it has attracted interest from scholars in this area, since its reduction is strongly correlated with postoperative complications, length of hospital stay, loss of functional status and death in the period of one year^18^.

In the present study, when evaluated the relation between MD and age and gender, the difference between the groups was not significant. However, Budziarecket et al.,^6^ who evaluated the influence of variables such as age and gender in healthy individuals, showed that age and gender have influenced muscle strength. It is worth mentioning that, until now, there are no reference values for MD in cancer patients in the literature.

The nutritional status of the patients studied, according to BMI, showed the prevalence of overweight and obesity (58.3%). The minority (16.7%) was underweight and this group presented a lower HS than the reference values for the healthy Brazilian population^6^. Likewise, Norman et al.^18^ found in their study that HS values were 25.8% lower in patients with low weight when compared to well nourished hospitalized patients and that MD was not significantly different in obese and eutrophic individuals.

Norman et al.^19^, using a sample of 189 patients with several solid cancers, observed that malnutrition, age and gender are contributing factors to the reduction of peripheral muscle strength in oncology patients and that the muscular strength evaluated with the assistance of the manual dynamometer was associated with the functional status and the quality of life, as they were expected to present similar determinants. The volume of medications per day, staging, tumor location and type of treatment did not affect the muscular strength of this population. The muscular strength measured by the manual dynamometry can be considered as a predictor of global muscle strength^25^.

Regarding the functionality, the surgical intervention caused alterations in its performance, presenting a significant loss when comparing the preoperative period and the 2^nd^ POD, and also an increase when comparing the 2^nd^ and the 7^th^ POD. Similar data were found in the study developed by Shida et al.^27^ that evaluates the physical independence through the Quality of Recovery Score (QoR-40) in patients with colorectal cancer who underwent surgical procedure, an opportunity that demonstrated an improvement of the functionality on the 6^th^ POD when compared to the immediate postoperative.

In the study developed by Santos et al.^24^, using a sample of 55 oncological patients who underwent surgical procedure, it was identified that the most impaired activities in the postoperative period were bed transfers and sleep. In this study, 83.3% and 33.4% of the patients needed the help of third parties (professional or companion) for the decubitus changes and ambulation, respectively, on the 2^nd^ POD. There was an improvement in mobility and transfer on the 7^th^ POD when compared to the 2^nd^ POD.

In the present study, the patients’ stay for more than 48 h in the ICU or postoperative unit occurred due to several reasons: hemodynamic changes, complications caused by the surgical procedure, respiratory changes and the need to maintain mechanical ventilation. The reasons that led the patients to the ICU admission mean that they need prolonged use of specific medications for each type of alteration and, in most cases, prolonged use of mechanical ventilation, factors that generate loss of the muscle strength and interfere in the functionality^3^. The patients in ICUs have limited control and influence on the environment, compromising both the psychic and functional factors. Thus, it was necessary to exclude this group to observe the result as exclusively related to the surgical procedure and hospital admission, without the intervention of other variables.

Therefore, the postoperative physical-functional recovery depends on several factors, including the resolution of pain and fatigue. This is related to altered sleep in the hospital environment, loss of muscle strength and weight loss^15^. The reduction of surgical stress, enteral nutrition and early mobilization are important interventions that counteract the fatigue and the need for assistance in basic activities^30^.

The pain reduction and the monitoring of the physiotherapy team may have influenced the increase in functionality on the 7^th^ POD, considering that the mobilization of the patient on the first postoperative day of high gastrointestinal resection aims to accelerate the physical-functional recovery and reduce the postoperative and pulmonary complications^10^. The bed rest is undesirable as it accelerates the mass loss and the muscle weakness and impairs lung function^30^, and also a prolonged bed rest can lead to other physical damage. The signs and the symptoms appear and can be observed in locomotion and in organic systems^2^ ^,^ ^30^.

There is no standard definition for early mobilization^8^. Brooks-Brunn^5^ using a sample of 400 patients undergoing abdominal surgery found that ambulation seemed to be the most beneficial activity in the immediate postoperative period showing evidences of improvements in lung function, prevention of functional decline and a positive effect on depression and anxiety^7^.

The limitation of this study was the small sample. Thus, many associations were not significant, and the possible explanations are based on the small number of patients studied.

## CONCLUSION

An important reduction in the handgrip strength and functionality was evidenced during the postoperative period in relation to the basal value in the pre-operative period.
